# COVID-19 Cases Among Congregate Care Facility Staff by Neighborhood of Residence and Social and Structural Determinants: Observational Study

**DOI:** 10.2196/34927

**Published:** 2022-10-04

**Authors:** Huiting Ma, Kristy C Y Yiu, Stefan D Baral, Christine Fahim, Gary Moloney, Dariya Darvin, David Landsman, Adrienne K Chan, Sharon Straus, Sharmistha Mishra

**Affiliations:** 1 St Michael's Hospital Unity Health Toronto Toronto, ON Canada; 2 Department of Epidemiology Johns Hopkins School of Public Health Baltimore, MD United States; 3 Division of Infectious Diseases Department of Medicine University of Toronto Toronto, ON Canada; 4 Dalla Lana School of Public Health University of Toronto Toronto, ON Canada; 5 Division of Infectious Diseases Sunnybrook Health Sciences University of Toronto Toronto, ON Canada; 6 Institute of Health Policy, Management and Evaluation University of Toronto Toronto, ON Canada; 7 Department of Medicine University of Toronto Toronto, ON Canada

**Keywords:** long-term care, nursing home, staff, essential worker, retirement home, shelter, congregate living, COVID-19, observational, risk, transmission, elderly, older adults, retirement, nurse, health care worker, congregate, trend, geography, Canada, Toronto

## Abstract

**Background:**

Disproportionate risks of COVID-19 in congregate care facilities including long-term care homes, retirement homes, and shelters both affect and are affected by SARS-CoV-2 infections among facility staff. In cities across Canada, there has been a consistent trend of geographic clustering of COVID-19 cases. However, there is limited information on how COVID-19 among facility staff reflects urban neighborhood disparities, particularly when stratified by the social and structural determinants of community-level transmission.

**Objective:**

This study aimed to compare the concentration of cumulative cases by geography and social and structural determinants across 3 mutually exclusive subgroups in the Greater Toronto Area (population: 7.1 million): community, facility staff, and health care workers (HCWs) in other settings.

**Methods:**

We conducted a retrospective, observational study using surveillance data on laboratory-confirmed COVID-19 cases (January 23 to December 13, 2020; prior to vaccination rollout). We derived neighborhood-level social and structural determinants from census data and generated Lorenz curves, Gini coefficients, and the Hoover index to visualize and quantify inequalities in cases.

**Results:**

The hardest-hit neighborhoods (comprising 20% of the population) accounted for 53.87% (44,937/83,419) of community cases, 48.59% (2356/4849) of facility staff cases, and 42.34% (1669/3942) of other HCW cases. Compared with other HCWs, cases among facility staff reflected the distribution of community cases more closely. Cases among facility staff reflected greater social and structural inequalities (larger Gini coefficients) than those of other HCWs across all determinants. Facility staff cases were also more likely than community cases to be concentrated in lower-income neighborhoods (Gini 0.24, 95% CI 0.15-0.38 vs 0.14, 95% CI 0.08-0.21) with a higher household density (Gini 0.23, 95% CI 0.17-0.29 vs 0.17, 95% CI 0.12-0.22) and with a greater proportion working in other essential services (Gini 0.29, 95% CI 0.21-0.40 vs 0.22, 95% CI 0.17-0.28).

**Conclusions:**

COVID-19 cases among facility staff largely reflect neighborhood-level heterogeneity and disparities, even more so than cases among other HCWs. The findings signal the importance of interventions prioritized and tailored to the home geographies of facility staff in addition to workplace measures, including prioritization and reach of vaccination at home (neighborhood level) and at work.

## Introduction

Across Canada, the COVID-19 epidemic has been marked by a conflation of microepidemics across settings, including congregate living facilities (eg, long-term care homes [LTCHs], retirement homes, and shelters), essential workplaces, or households [1-6]. Many congregate facilities experienced outbreaks, with residents experiencing a 3- to 5-fold higher test positivity rate than the community-dwelling population [1,3,4,7,8]. Meanwhile, data on the community-dwelling population suggested a consistent pattern of geographic clustering of cases with 50% of cases occurring in approximately 25% of the population and disproportionately affecting those residing in neighborhoods that are less affluent and with a higher proportion of essential workers [9,10].

What is less understood is how congregate facilities may be connected with neighborhood disparities. Emerging data on congregate facility outbreaks suggested that facility-level spread among staff, among residents, and between staff and residents may have been partially triggered by undiagnosed infections and lack of support for effective isolation among staff [3,7]. Reducing transmission in the wider community may reduce outbreaks in congregate settings. However, data on facility staff are limited with few studies that characterized the socioeconomic, living, and working conditions of staff [11] and, thus, the extent to which social and structural determinants of community-level transmission might influence outbreaks in congregate facilities. Surveillance data on COVID-19 cases offer an opportunity to examine the pattern of cases among facility staff and other health care workers (HCWs) against community cases and neighborhood disparities. Lorenz curves, Gini coefficients, and the Hoover index, traditionally used as measurements of economic inequality [12,13], have been used in a range of health care research to measure health inequality [14,15]; for example, these measurements of health inequality have been recently applied in the context of the COVID-19 pandemic [10,16]. We sought to adapt Lorenz curves, Gini coefficients, and the Hoover index to compare the concentration of cases using neighborhood-level rates and neighborhood-level social and structural determinants across 3 mutually exclusive subgroups: community, facility staff (LTCHs, retirement homes, shelters), and other HCWs not working in congregate settings (eg, only working in hospitals). Our overarching objective was to determine if and how the pattern and magnitude of inequality and concentration in COVID-19 cases among HCWs differed between facility staff and other HCWs.

## Methods

### Study Design, Setting, and Population

We conducted a retrospective, observational study using provincial surveillance data on laboratory-confirmed COVID-19 cases reported between January 23, 2020, and December 13, 2020, in the Greater Toronto Area (population: 7.1 million) [17] and in accordance with the RECORD (Reporting of Studies Conducted Using Observational Routinely-Collected Data) statement [18]. We restricted the study to the period before COVID-19 vaccination was available due to differential vaccine allocation and coverage over time by each subgroup after vaccine rollout [19].

### Data Sources and Measures

We used person-level data from Ontario’s centralized surveillance system [20], which includes information on laboratory-confirmed COVID-19 cases by reported date, demographic characteristics, exposure category, and setting-specific characteristics (eg, LTCHs), as well as data on social and structural determinant measures from the Statistics Canada 2016 Census [21]. The surveillance data classify cases as an HCW if a person works or volunteers in any health care setting (including LTCHs, retirement homes, shelters, hospitals, clinics, or homecare). We stratified HCWs into those associated with working or volunteering in an LTCH, retirement home, and/or shelter as facility staff, and all others as “other HCWs.” If an HCW fell into both categories (facility staff and other HCWs), then they were categorized as facility staff.

We examined social and structural determinants at the level of the dissemination area (neighborhood) because it was the smallest geographic unit (population size ranging from 400 to 700) for which census data were available. Other geographic units include the forward sortation area and census tracts, but the dissemination area is most commonly used when examining social and structural determinants because it reflects the smallest geographic unit and is less prone to ecological fallacy than larger geographic units [22]. We conceptualized and defined the social and structural determinants as reported previously [9,10]. The variables are detailed in Multimedia Appendix 1 [21,23,24] and are related to socioeconomic status (per-person equivalent after tax income) and proxies for systemic racism (% visible minority, % recent immigration), or to the potential for increased contact rates (housing: % not living in high-density housing [25,26], % living in multigenerational households) and employment in other essential services (ie, excluding health care) [27] not amenable to remote work [28].

### Analyses

We aggregated the number of confirmed COVID-19 cases at the neighborhood level during the study period into 3 mutually exclusive subgroups: community (excluding facility staff, other HCWs, congregate facility residents, and travel-related cases), facility staff (workers or volunteers in LTCHs, retirement homes, and shelters), and other HCWs. We generated Lorenz curves and Gini coefficients to quantify the magnitude of inequalities (ie, the concentration in cases), and the Hoover index was used as an alternate measure for validation [15,16,29]. With the Gini coefficient, a value closer to zero represents greater equality [30]. The Hoover index measures the percentage of cases that would need to be redistributed to achieve equality in how cases are distributed across neighborhoods. As with the Gini coefficient, a larger Hoover index represents greater inequality [31]. We generated 95% CIs for Gini coefficients using bootstrapping [29,32].

First, we compared the magnitude of geographic concentration of cases for each subgroup (y-axis) against the distribution of total cases (x-axis, community plus travel related) at the neighborhood level. To examine the extent to which facility staff and other HCW cases mirrored community cases, we generated a separate set of Lorenz curves and Gini coefficients using community cases on the x-axis. Second, to examine the magnitude of inequalities by each social or structural determinant, we ranked the cumulative proportion of the population by each determinant (eg, from lowest to highest income decile) on the x-axis. A detailed analytic plan can be found in Multimedia Appendix 2 [9,10,15,16,20-22,25-32]. We also generated spatial maps to describe and overlay cases among facility staff and among other HCWs using one social determinant as an example (neighborhood-level income).

Analyses were conducted in R (version 4.0.2; R Core Team), and spatial maps were generated using ArcGIS (version 10.7; Esri).

### Ethics Approval

The University of Toronto Health Sciences Research Ethics Board approved the study (protocol #39253).

## Results

### Overview

Of the 92,210 cases (excluding congregate facility residents and travel-related cases) included during our study period, there were 83,419 cases in the community, 4849 cases among facility staff, and 3942 cases among other HCWs (Table 1). Among facility staff, there were 4241, 363, and 245 cases among LTCH staff, retirement home staff, and shelter staff, respectively.

**Table 1:** Number of COVID-19 cases in 3 mutually exclusive subgroups (community, facility staff, and other health care workers) in the Greater Toronto Area (January 23, 2020, to December 13, 2020).

Subgroup	COVID-19 cases, n	Dissemination areas^a^ with zero cases, n (%)^b^
Community^c^	83,419	1058 (12.8)
Facility staff^d^	4849	5771 (69.7)
Other health care workers	3942	5879 (71.0)

^a^Dissemination area refers to the geographic unit of measurement for the social and structural determinants examined in this study generated from Statistics Canada [21]. In the Greater Toronto Area (population: 7.1 million), the median population size of a dissemination area is 561 (IQR 442-800) residents.

^b^A total of 8278 dissemination areas in the region.

^c^Excludes residents of congregate settings and facility staff (long-term care homes, retirement homes, and shelters), other health care workers, and travel-related cases.

^d^Includes staff and volunteers who work in long-term care homes, retirement homes, and shelters, and excludes all other health care workers.

### Geographic Concentration of Cases Across Subgroups

**Figure 1:**
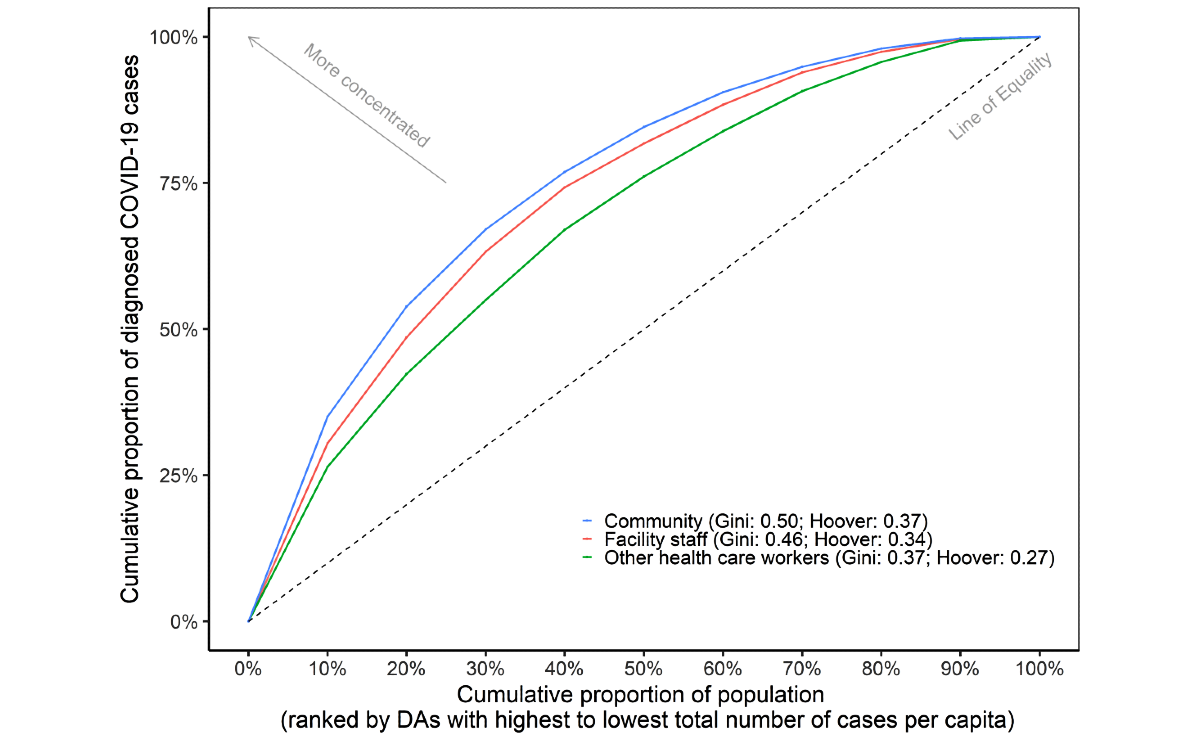
Geographic concentration of COVID-19 cases in the community population, among facility staff, and among other health care workers in the Greater Toronto Area (January 23, 2020, to December 13, 2020). The magnitude of concentration is depicted by Lorenz curves (the dashed line represents the line of equality) and the corresponding Gini coefficient for each subgroup. The x-axis represents the cumulative proportion of the population ranked by DAs from the highest to lowest number of cumulative cases per capita. “Community” excludes residents of congregate settings and facility staff (long-term care homes, retirement homes, and shelters), other health care workers, and travel-related cases. “Facility staff” includes staff and volunteers who work in long-term care homes, retirement homes, and shelters and excludes all other health care workers. DA: dissemination area.

The most affected neighborhoods (x-axis) comprising 20% of the total population accounted for 53.87% (44,937/83,419) of community cases, 48.59% (2356/4849) of congregate setting worker cases, and 42.34% (1669/3942) of other HCW cases (Figure 1). Compared with other HCWs, cases among facility staff more closely reflected the geographic distribution of community cases (Gini 0.06 vs 0.16; Hoover 0.05 vs 0.12) (Multimedia Appendix 3).

### Differences in the Concentration of Cases Across Subgroups by Social and Structural Determinants

**Figure 2:**
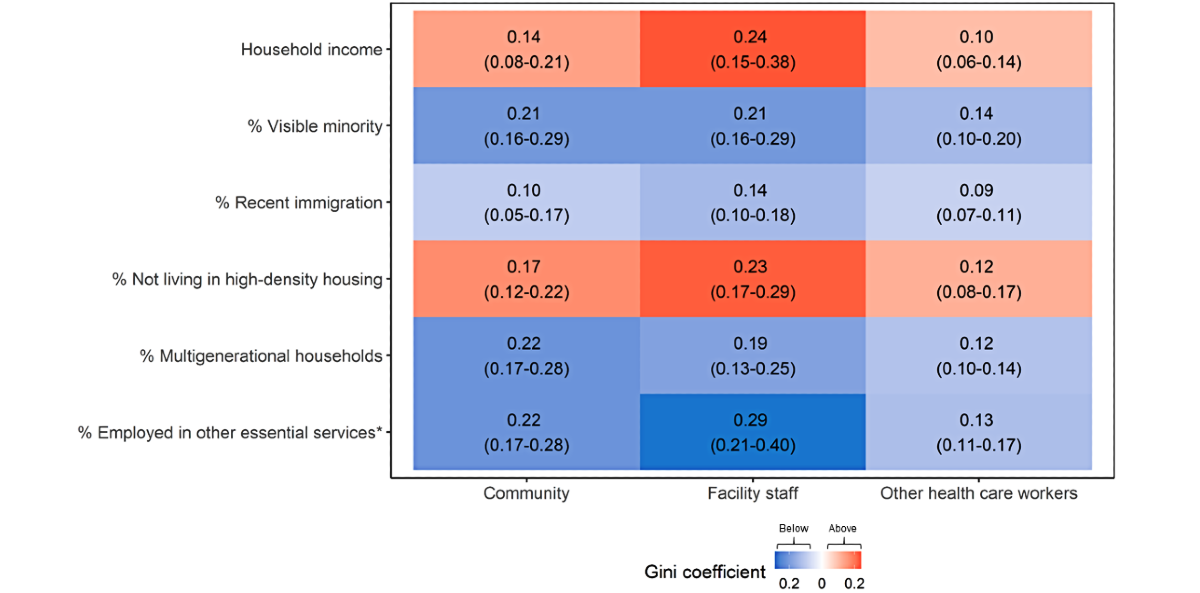
A heat map with estimated Gini coefficients showing the magnitude of concentration by social and structural determinants in COVID-19 cases in the community, among facility staff, and among other health care workers in the Greater Toronto Area (January 23, 2020, to December 13, 2020). Gini coefficients above and below the line of equality in the Lorenz curves (Multimedia Appendix 5) are depicted in red and blue, respectively. *Other essential services include trades, transport, and equipment operation; sales and services; manufacturing and utilities; and resources, agriculture, and production [32]. “Community” excludes residents of congregate settings and facility staff (long-term care homes, retirement homes, and shelters), other health care workers, and travel-related cases. “Facility staff” includes staff and volunteers who work in long-term care homes, retirement homes, and shelters and excludes all other health care workers.

Multimedia Appendix 4 depicts Lorenz curves and Gini coefficients by each social and structural determinant. Cases among facility staff reflected greater social and structural inequalities (larger Gini coefficients and a larger Hoover index) than other HCWs across all determinants (Figure 2, Multimedia Appendices 4 and 5). Multimedia Appendix 6 depicts how cases among facility staff and other HCWs were clustered along neighborhood-level income. Although facility-level cases mirrored that of community cases (Multimedia Appendix 3), there were greater inequalities in facility-level versus community cases with respect to income (Figure 2; Gini 0.24, 95% CI 0.15-0.38 vs 0.14, 95% CI 0.08-0.21), household density (Gini 0.23, 95% CI 0.17-0.29 vs 0.17, 95% CI 0.12-0.22), and other essential services (Gini 0.29, 95% CI 0.21-0.40 vs 0.22, 95% CI 0.17-0.28). Cases in the community, among facility staff, and among other HCWs were disproportionately concentrated in areas with lower household income and in areas with a higher proportion of visible minorities, recent immigration, high-density housing, multigenerational households, and employment in other essential services (Figure 2).

## Discussion

We found that the distribution of COVID-19 cases among facility workers mirrored neighborhood heterogeneity and social and structural disparities, a pattern that was less evident with other HCWs. Facility staff cases reflected greater inequalities by social and structural determinants than cases among other HCWs, and with some determinants (income and other essential workers), greater inequalities were seen with facility staff cases compared to community cases.

Cases among facility staff more closely reflected the geographic distribution of community cases than the distribution of cases among other HCWs and in the community. The similar distributions of facility staff and community cases could occur if there was an equal distribution of facility staff living across neighborhoods and the infection rate ratio between facility staff and the community was the same across neighborhoods. The implication of this potential mechanism is that most infections among facility staff would have been acquired outside the facility or workplace. The Lorenz curve patterns may also occur if facility staff were more likely than other HCWs to live in harder-hit neighborhoods with greater social and structural disparities, irrespective of workplace exposures in congregate facilities.

Although our study was centered on the Greater Toronto Area, the findings are likely to be generalizable to other large, urban cities and metropolitan areas with similar patterns of social and structural inequalities. In Canada, most congregate facilities are concentrated in large cities [34], and previous research comparing 16 cities demonstrated a similar neighborhood clustering of COVID-19 cases by social and structural determinants [10]. Data on the neighborhood characteristics of LTCH staff in the United States [11] suggest workers tend to live in lower-income neighborhoods, and individual-level data in Canada suggest that a high proportion of LTCH staff identify as racialized women with low household income [35]. In current LTCH staffing models across Canada and the United States, approximately 60% to 90% of workers who provide direct care to residents are providing services as unregulated staff (personal support workers, care aides, orderlies, and nurse assistants) [36-40] and receive the lowest wages in the health care sector at or just above minimum wage [36], often in the context of contract or casual work without benefits [36,37,41-45].

Our study suggests that cases among facility staff may disproportionately intersect with household exposures that are connected with other essential workplaces or amplified in the context of household density [21]. Our study was limited by a lack of confirmed denominators for setting-specific workers in the region, but data from England and Wales suggest a 2-fold increased rate of COVID-19 among LTCH workers versus other HCWs [46]. Based on government reports, just over 100,000 employees serve 78,000 LTCH residents in the province [36], such that the ratio of staff to LTCH residents in Ontario is approximately 1 staff for every 0.78 LTCH resident. If we extrapolate the provincial ratio to the Greater Toronto Area, where 28,316 LTCH residents reside, the city would have approximately 36,303 LTCH staff. With 4241 cases among LTCH staff in the Greater Toronto Area during our study period and a total population in the Greater Toronto Area of 7.1 million [17], the cumulative rate of COVID-19 cases among LTCH staff (at 11,682 per 100,000) would have been 10 folds higher than that of the community (1174 cases per 100,000).

This study has several limitations. First, we derived the dissemination area–level social determinants from the 2016 Census data, which may not be representative of the population during the COVID-19 pandemic. Second, the occupation status obtained from Ontario’s centralized surveillance system could have been misclassified due to possible misinterpretation of the question in self-reporting; further, facility staff or HCWs may work in multiple settings. Third, we did not have data on the residence of all HCWs across the various congregate settings to compare neighborhood-level per-HCW rates of cases. Finally, data were not available to link cases among HCWs to specific facilities and to directly examine how cases in communities influenced outbreaks in congregate settings.

The findings have implications for COVID-19 modeling and interventions. The magnitude of inequalities can be used as calibration or validation targets for epidemic and prediction models to reproduce the observed pattern of cases in relation to the distribution of overall cases and by social and structural determinants. In doing so, detection systems (eg, neighborhood wastewater surveillance) designed to predict the potential for exposures in congregate facilities could leverage data on underlying vulnerabilities in the neighborhood of facility staff residence. A study in the United States found that neighborhood characteristics of LTCH staff’s residences were the most important predictor of LTCH outbreaks [11]. These data could then be used to implement strategies to mitigate risks. For example, proximal strategies to reduce community-level transmission risks conferred through social and structural inequalities have the potential to reduce workplace exposure risks. Examples include systematically addressing the lived realties of workers that make physical distancing challenging (eg, household density) or that remain barriers to effective isolation and quarantine (eg, precarious job security and absence of benefits such as paid sick leave), with interventions such as wraparound care including access to food, medications, and child and senior care (especially in the context of multigenerational households) to facilitate staff quarantine and isolation. Prioritizing vaccination coverage in the hardest-hit neighborhoods is another example of indirectly reducing workplace exposures in LTCHs, retirement homes, and shelters. Finally, the findings highlight an urgent need for a long-term commitment and resources to comprehensively address social and structural barriers at a systems level (integration of health, education, social services, public health, and labor) given the long-standing history of infectious disease outbreaks in facilities and disparities experienced by its staff even before the COVID-19 pandemic [37].

## Appendix

Multimedia Appendix 1 Social determinants of health: variables from the Statistics Canada 2016 Census of Population.

Multimedia Appendix 2 Detailed analytic plan.

Multimedia Appendix 3 Geographical concentration of COVID-19 cases among facility staff and other health care workers compared with community cases in the Greater Toronto Area, from January 23, 2020, to December 13, 2020.

Multimedia Appendix 4 Lorenz curves and Gini coefficients of COVID-19 cases in the community, among facility staff, and among other health care workers by social determinants in the Greater Toronto Area, from January 23, 2020, to December 13, 2020.

Multimedia Appendix 5 Magnitude of concentration of COVID-19 cases by social and structural determinants in the community, among facility staff, and among other health care workers in the Greater Toronto Area, from January 23, 2020, to December 13, 2020.

Multimedia Appendix 6 Map overlay of household income deciles by dissemination area and distribution of (A) COVID-19 facility staff cases and (B) other health care worker cases in the Greater Toronto Area, from January 23, 2020, to December 13, 2020.

## Abbreviations

HCW: health care worker
LTCH: long-term care home
RECORD: Reporting of Studies Conducted Using Observational Routinely-Collected Data

## Data Availability

Reported COVID-19 cases were obtained from Case and Contact Management Solutions via the Ontario COVID-19 Modelling Consensus Table and with approval from the University of Toronto Health Sciences Research Ethics Board. The analyses, conclusions, opinions, and statements expressed herein are solely those of the authors and do not reflect those of the funding or data sources; no endorsement is intended or should be inferred.
