# Supplementary material for: COVID-19 Cases Among Congregate Care Facility Staff by Neighborhood of Residence and Social and Structural Determinants: Observational Study
Source: JMIR Public Health Surveill. 2022 Oct 4;8(10):e34927. doi: 10.2196/34927 (PMC9534317; doi:10.2196/34927)
Supplement: Multimedia Appendix 1 [file publichealth_v8i10e34927_app1.docx]

*Appendix 1. Social determinants of health – Variables from Statistics Canada 2016 Census of Population*

| Measure (*Source*)^a^ | Definition of indicator | Notes^b^ [21, 23] |
| --- | --- | --- |
| Population size  (100% *of census sample*) | Total population count of a Dissemination Area | In this measure and where required, Dissemination Area population counts are adjusted (reduced) to remove facility-staff (long-term care homes^c^, retirement homes, and persons using shelters), and travel-related cases. |
| **Socio-demographic** |  |  |
| Household income  (*100% of census sample*)^d^ | Decile rank of a Dissemination Area’s average total after-tax income, weighted by population | After-tax income is calculated for each household from the income for all household members. Calendar year 2015 is the reference period for all income variables in the 2016 Census. Single-person equivalent is used to account for households of different sizes. To limit variations in the cost of living, the ranking is calculated exclusively from DAs within the City of Toronto. |
| % recent immigration  (*25% of census sample*) | Numerator: Number of persons who immigrated to Canada in the 5 year period between 2011 and 2016  Denominator: Total population within the Dissemination Area | 2016 Census Dictionary states: 'Immigrant' refers to a person who is, or who has ever been, a landed immigrant or permanent resident. Such a person has been granted the right to live in Canada permanently by immigration authorities.  2016 Census Dictionary states: 'Period of immigration' refers to the period in which the immigrant first obtained landed immigrant or permanent resident status. |
| % visible minority  (*25% of census sample*) | Numerator: Number of persons who belong to visible minority groups  Denominator: Total population within the Dissemination Area | Visible minority groups are defined by the Employment Equity Act: "persons, other than Aboriginal peoples, who are non-Caucasian in race or non-white in colour". 2016 Census Dictionary states: “The visible minority population consists mainly of the following groups: South Asian, Chinese, Black, Filipino, Latin American, Arab, Southeast Asian, West Asian, Korean and Japanese.” |
| **Dwelling-related** |  |  |
| % not living in high-density housing  (*25% of census sample*) | Numerator: Number of private households^e^ living in dwellings that have “enough bedrooms for the size and composition of the household.” [23]  Denominator: Total number of private households within the Dissemination Area | The National Occupancy Standard (NOS) is used to classify the suitability of accommodations. A suitable household (not high-density housing) is defined as "households where the required number of bedrooms based on the National Occupancy Standard (NOS) does not exceed the reported number of bedrooms in the dwelling.” The number of required bedrooms is determined using the following criteria [24]:  1. A maximum of two persons per bedroom.  2. Household members, of any age, living as part of a married or common-law couple share a bedroom with their spouse or common-law partner.  3. Lone-parents, of any age, have a separate bedroom.  4. Household members aged 18 or over have a separate bedroom - except those living as part of a married or common-law couple.  5. Household members under 18 years old of the same sex share a bedroom - except lone-parents and those living as part of a married or common-law couple.  6. Household members under 5 years old of the opposite sex share a bedroom if doing so would reduce the number of required bedrooms. This situation would arise only in households with an odd number of males under 18, an odd number of females under 18, and at least one female and one male under the age of 5. |
| multigenerational households  (*100% of census sample*) | Numerator: Number of persons who live in households where “at least one person [in the household is] living with a child and a grandchild.” [23]  Denominator: Total of all persons who are classified by family status and household living arrangements | This measure is a count of persons whose households are described by a specific living arrangement. All persons in the same household are counted separately. In the numerator for this measure, family status includes persons who are married spouses, common-law partners, lone parent families, and the child(ren) of these persons. Persons living alone, with other relatives, or with non-relatives only are additionally included in the denominator count of persons. Couples can be opposite or same sex. |
| **Occupation-related** |  |  |
| % other essential services not amenable to remote working  (25% *of census sample*) | Numerator: Number of persons in the labor force who have occupations in one of the following categories: Manufacturing/utilities, Trades/transport/equipment operators, Sales/services, Resources/agriculture/production  Denominator: Total labor force population aged 15 years and over in private households in the Dissemination Area | Occupations are assigned according to the National Occupancy Classification (2016). Occupation was chosen over “Industry” to better represent the type of work performed and skill-level required by a population rather than the industry that provides the employment. Numerators may be defined separately (“or”) or added together in different combination sets (“and”). “Labor Force” is all persons in private households aged 15 years and older who were either employed or unemployed during the week of Sunday, May 1 to Saturday, May 7, 2016. |

^a^ “Sample” refers to the short-form Census questionnaire (*100%* sample) or to the long-form questionnaire, received by a random sample of households (*25%* sample). It is mandatory for recipients to respond to the questionnaires. Statistical inferences for the entire population are drawn from the subset of responses of the long-form questionnaire; these inferences are reported in the tabulated values provided by Statistics Canada. Note that income information was collected solely from administrative data sources (*100%* sample) and were not part of either questionnaire.

^b^Additional details about variable definitions may be included the Census Dictionary; please refer to Statistics Canada’s Dictionary for the 2016 Census of Population for complete definitions. Some definitions provided here are taken verbatim from source.

^c^ Due to reporting methods used by CCM+, case counts among “Long-Term Care Residents” may also include cases that are reported for residents of “nursing home[s] or other chronic care facility[ies]”. Adjustments in population counts described here only include adjustments to Dissemination Areas that have one (or more) LTCH facility identified by the Ontario Ministry of Health. The adjustments are made by subtracting the total number of beds in the facility from the population count of the DA.

^d^ Income deciles for the City of Toronto / Toronto Public Health Unit were tabulated by ICES from data contained in PCCF+ (version 7B) and adjusted for population size. **Ref: Statistics Canada. 2018. Postal Code Conversion File Plus (PCCF+) Version 7B, Reference Guide. November 2018 Postal codes**.

^e^ Where referenced, “household” refers to a “private household”. The 2016 Census Dictionary states: ”Private household” refers to a person or group of persons who occupy the same dwelling and do not have a usual place of residence elsewhere in Canada or abroad.
