# Supplementary material for: COVID-19 Cases Among Congregate Care Facility Staff by Neighborhood of Residence and Social and Structural Determinants: Observational Study
Source: JMIR Public Health Surveill. 2022 Oct 4;8(10):e34927. doi: 10.2196/34927 (PMC9534317; doi:10.2196/34927)
Supplement: Multimedia Appendix 2 [file publichealth_v8i10e34927_app2.docx]

*Appendix 2. Detailed analytic plan*

*Data sources and measures*

We used person-level data from the provincial surveillance system [20] which includes information on laboratory-confirmed COVID-19 cases by reported date, demographic characteristics, exposure category, and setting specific characteristics (e.g., long-term care home [LTCH]), and data on social and structural determinant measures from Statistics Canada 2016 Census [21]. We used the variables “LTCH_HCW”, “OCC_LTCH”, “OCC_RETIREMENTHOME”, and “OCC_SHELTERHOMELESSSTAFF” from the provincial surveillance system to identify workers in LTCH, retirement homes, and shelters.  The surveillance data classifies cases as a health-care worker (HCW) if a person works or volunteers in any health-care setting (including LTCH, retirement home, shelter, hospital, clinic, or homecare). We stratified HCW into those associated with working/volunteering in a LTCH, retirement home, and/or shelter as facility-staff; and all others as “other HCW”. If a HCW fell into both categories (facility-staff and other HCW), then they were categorized as facility-staff.

We examined social/structural determinants at the level of the dissemination area (neighbourhood) because it was the smallest geographic unit (range of population size 400 to 700) for which census data were available. Other geographic units include the forward sortation area and census tracts, but the dissemination area is most commonly used when examining social/structural determinants because it reflects the smallest geographic unit and is less prone to ecological fallacy than larger geographic units [22]. We conceptualized and defined the social and structural determinants as reported previously [9, 10]. The variables are detailed in *Appendix 1* and are related to socio-economic status (per-person equivalent after tax income) and proxies for systemic racism (% visible minority, % recent immigration); or to the potential for increased contact rates: housing (% not living in high-density housing [25, 26], % living in multigenerational households) and employment in other essential services (i.e. excluding healthcare) [27] not amenable to remote work [28].

*Analyses*

First, we aggregated the number of confirmed COVID-19 cases at the neighbourhood-level during the study period into the following three mutually exclusive subgroups: community (excluding facility-staff, other health-care workers (HCW), congregate-facility residents, and travel-related cases), facility-staff (workers in LTCH, retirement homes, and shelters); and other HCW.

Second, we generated crude Lorenz curves,Gini coefficients to examine the relationship between two cumulative distributions, and the Hoover index as an alternate measure for validation [15,16,29]: the cumulative proportion of population after ranking the total community cases per-capita (community cases plus travel-related cases) at the neighbourhood-level by decile (x-axis) and the cumulative proportion of confirmed COVID-19 cases in each subgroup (y-axis) [30]. To examine the extent to which facility-staff and other HCW cases mirrored community cases, we generated a separate set of Lorenz curves and Gini coefficients using community cases in the x-axis.

Third, we investigated the concentration of cases at each subgroup by each social and structural determinant. We also generated spatial maps to describe and overlay cases among facility-staff and among other HCW, by one social determinant as an example (neighbourhood-level income). To generate the Lorenz curves and Gini coefficients, we plotted the cumulative proportion of population ranked in deciles (from the lowest to highest value [e.g. from the lowest to highest income decile]) on the x-axis and the corresponding cumulative proportion of confirmed cases in each subgroup on the y-axis [30].

The Gini coefficient is a summary measure of the inequality in the distribution of cases, where a value closer to zero represents greater equality and a value closer to one represent greater inequality [30]. The Hoover index measures the percentage of cases that would need to be redistributed to achieve equality in how cases are distributed across neighbourhoods. As with the Gini coefficient, a larger Hoover index represents greater inequality [31]. We generated 95% confidence interval for Gini coefficients using bootstrapping [29, 32].
