# Supplementary material for: COVID-19 Cases Among Congregate Care Facility Staff by Neighborhood of Residence and Social and Structural Determinants: Observational Study
Source: JMIR Public Health Surveill. 2022 Oct 4;8(10):e34927. doi: 10.2196/34927 (PMC9534317; doi:10.2196/34927)
Supplement: Multimedia Appendix 3 [file publichealth_v8i10e34927_app3.docx]

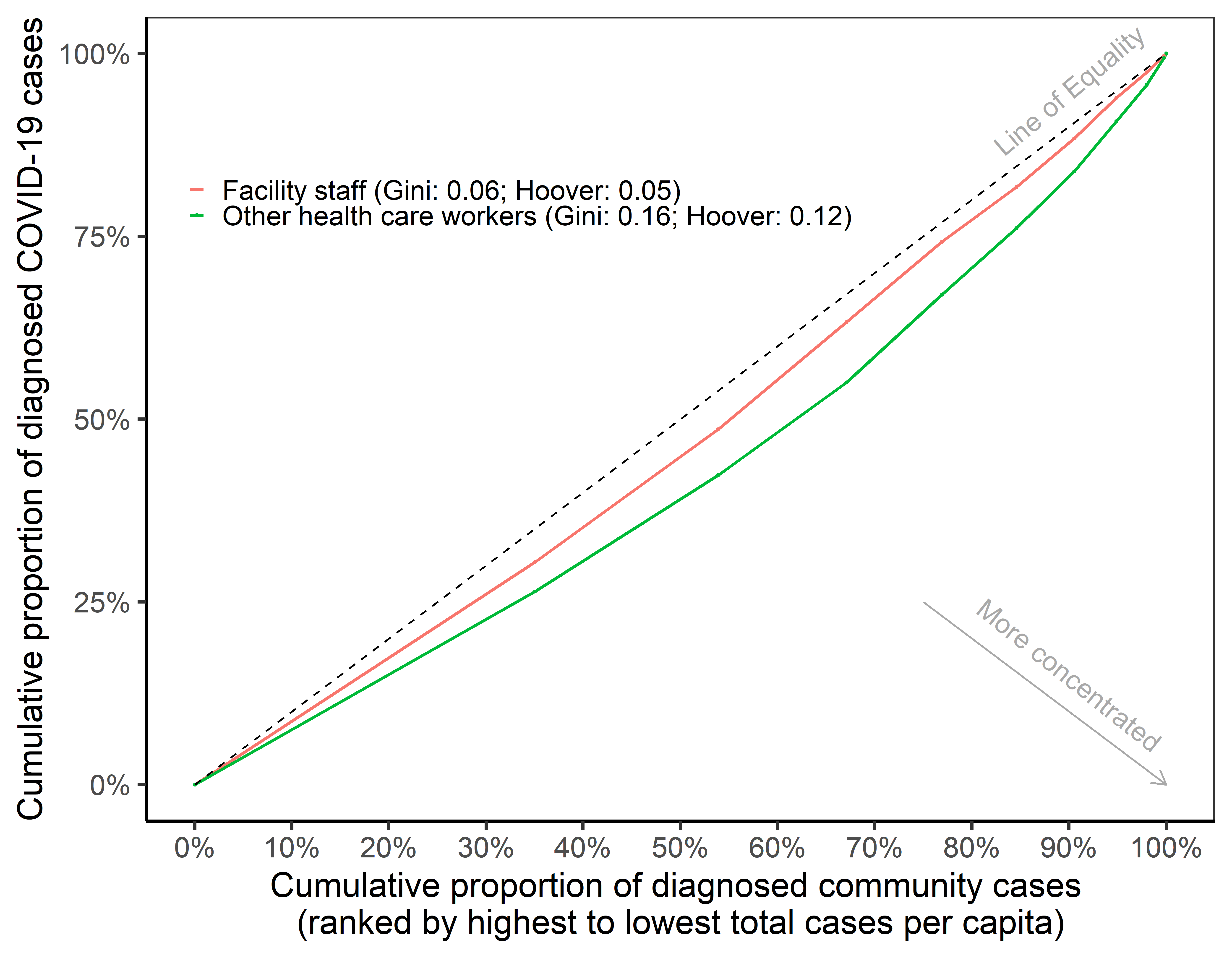


*Appendix 3. Geographical concentration of COVID-19 cases among facility staff, other health care workers compared with community cases in the Greater Toronto Area, from January 23, 2020 to December 13, 2020.* The magnitude of concentration is depicted by Lorenz curves (dashed line represents the line of equality), and the corresponding Gini coefficient for each subgroup. The x-axis represents the cumulative proportion of the diagnosed community cases ranked by dissemination areas from the highest to the lowest number of cumulative cases per capita. “Facility staff” includes and volunteers who work in long-term care homes, retirement homes, and shelters and excluding all other health care workers; “Community” excludes residents of congregate settings and facility staff (long-term care homes, retirement homes, and shelters), other health care workers, and travel-related cases.
