# Supplementary material for: COVID-19 Cases Among Congregate Care Facility Staff by Neighborhood of Residence and Social and Structural Determinants: Observational Study
Source: JMIR Public Health Surveill. 2022 Oct 4;8(10):e34927. doi: 10.2196/34927 (PMC9534317; doi:10.2196/34927)
Supplement: Multimedia Appendix 4 [file publichealth_v8i10e34927_app4.docx]

*Appendix 4. Lorenz curves and gini coefficients of COVID-19 cases in the community^a^, among facility staff^b^, and among other health care workers by social determinants in Greater Toronto Area, from January 23, 2020 to December 13, 2020.* The magnitude of concentration is depicted by Lorenz curves (dashed line represents the line of equality), and the corresponding Gini coefficient for each subgroup. The x-axis represents the cumulative proportion of the population ranked by dissemination areas with the lowest to highest percentage for each social determinant. “Community” excludes residents of congregate settings and facility staff (long-term care homes, retirement homes, and shelters), other health care workers, and travel-related cases; “Facility staff” includes staff and volunteers who work in long-term care homes, retirement homes, and shelters and excludes all other health care workers. Other essential services include: trades, transport and equipment operation; sales and services; manufacturing and utilities; resources, agriculture, and production. For example, dissemination areas with the lowest household income and account for 20% population in the Greater Toronto Area account for 26% of cases in the community, 31% of cases among facility staff, and 24% of cases among other health care workers, respectively.
