# Supplementary material for: COVID-19 Cases Among Congregate Care Facility Staff by Neighborhood of Residence and Social and Structural Determinants: Observational Study
Source: JMIR Public Health Surveill. 2022 Oct 4;8(10):e34927. doi: 10.2196/34927 (PMC9534317; doi:10.2196/34927)
Supplement: Multimedia Appendix 5 [file publichealth_v8i10e34927_app5.docx]

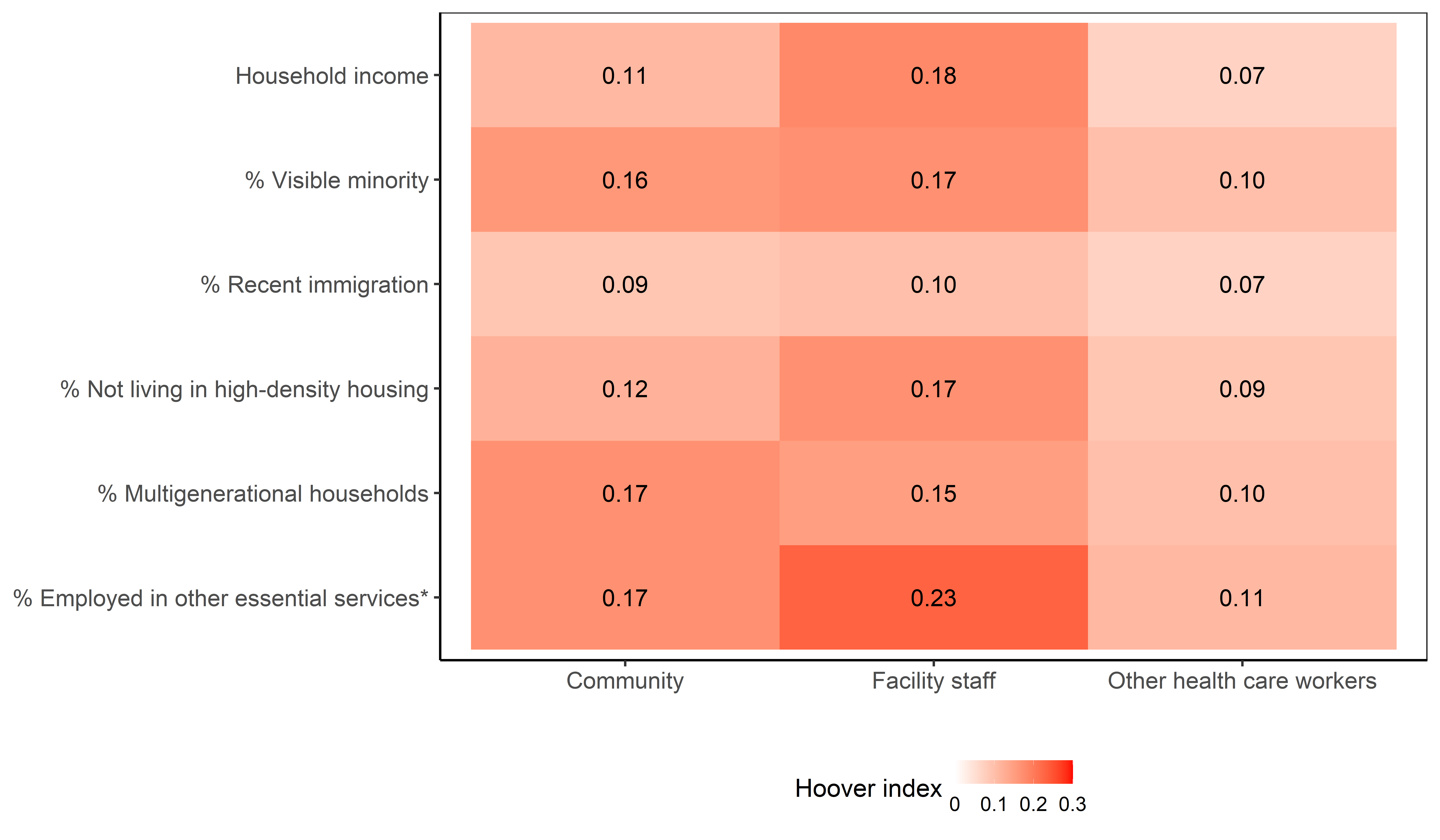


*Appendix 5. Magnitude of concentration by social and structural determinants in COVID-19 cases in the community, among facility staff, and among other health care workers in the Greater Toronto Area (January 23, 2020 to December 13, 2020).* Heat map with the estimated Hoover Index of cumulative COVID-19 cases by household income, % visible minority, % recent immigration, % not living in high-density housing, % multigenerational households, and % employed in other essential services. The Hoover Index represents the percentage of cases that require to be redistributed to achieve case concentration equity. Higher values on the Hoover Index are depicted in darker shades of red. ^*^Other essential services include: trades, transport and equipment operation; sales and services; manufacturing and utilities; resources, agriculture, and production [28]. “Community” excludes residents of congregate settings and facility staff (long-term care homes, retirement homes, and shelters), other health care workers, and travel-related cases. “Facility staff” includes staff and volunteers who work in long-term care homes, retirement homes, and shelters and excludes all other health care workers.
