# Supplementary material for: COVID-19 Cases Among Congregate Care Facility Staff by Neighborhood of Residence and Social and Structural Determinants: Observational Study
Source: JMIR Public Health Surveill. 2022 Oct 4;8(10):e34927. doi: 10.2196/34927 (PMC9534317; doi:10.2196/34927)
Supplement: Multimedia Appendix 6 [file publichealth_v8i10e34927_app6.docx]

(A)


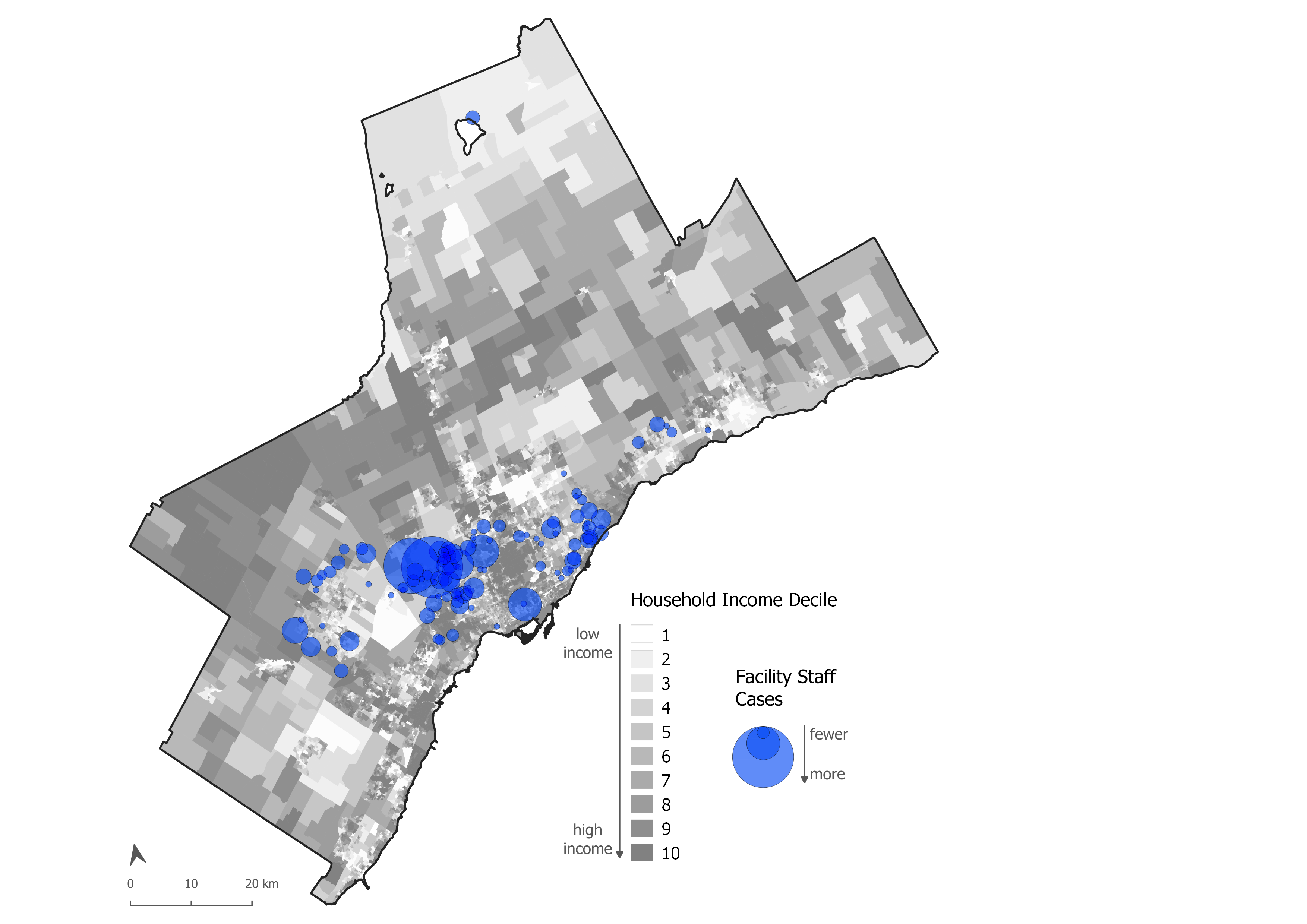


(B)


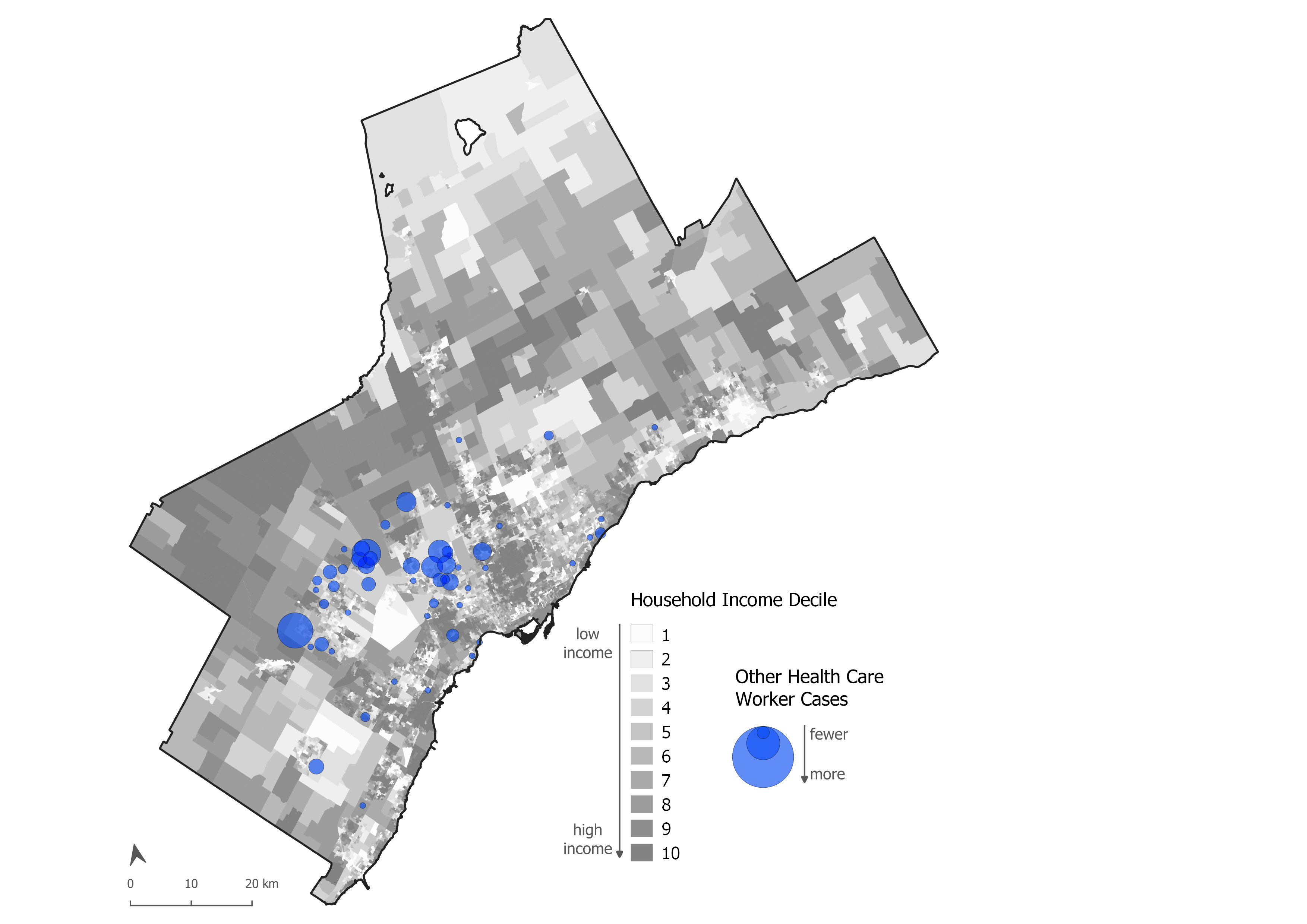


*Appendix 6. Map overlay of household income deciles by dissemination areas and distribution of COVID-19 facility staff (A) and other health care workers cases (B) in the Greater Toronto Area (January 23, 2020 to December 13, 2020).* “Facility staff” includes staff and volunteers who work in long-term care homes, retirement homes, and shelters and excludes all other health care workers (i.e., not working in congregate settings). The size of the circle is proportional to the number of cases.
